# Effects of Extrusion Pretreatment Parameters on Sweet Sorghum Bagasse Enzymatic Hydrolysis and Its Subsequent Conversion into Bioethanol

**DOI:** 10.1155/2015/325905

**Published:** 2015-03-19

**Authors:** Erick Heredia-Olea, Esther Pérez-Carrillo, Manuel Montoya-Chiw, Sergio O. Serna-Saldívar

**Affiliations:** Centro de Biotecnología FEMSA, Escuela de Ingeniería y Ciencias, Tecnológico de Monterrey, Avenida Eugenio Garza Sada 2501 Sur, 64849 Monterrey, NL, Mexico

## Abstract

Second-generation bioethanol production from sweet sorghum bagasse first extruded at different conditions and then treated with cell wall degrading enzymes and fermented with *I. orientalis* was determined. The twin extruder parameters tested were barrel temperature, screws speed, and feedstock moisture content using surface response methodology. The best extrusion conditions were 100°C, 200 rpm, and 30% conditioning moisture content. This nonchemical and continuous pretreatment did not generate inhibitory compounds. The extruded feedstocks were saccharified varying the biocatalysis time and solids loading. The best conditions were 20% solids loading and 72 h of enzymatic treatment. These particular conditions converted 70% of the total fibrous carbohydrates into total fermentable C5 and C6 sugars. The extruded enzymatically hydrolyzed sweet sorghum bagasse was fermented with the strain *I. orientalis* at 12% solids obtaining a yield of 198.1 mL of ethanol per kilogram of bagasse (dw).

## 1. Introduction

There are many pretreatments that can be used to prepare or obtain the fermentable sugars from lignocellulosic raw materials. The most popular pretreatments include acid hydrolysis, steam explosion, ammonia fiber expansion, alkaline wet oxidation, and hot water pretreatment [1, 2]. However, all the mentioned treatments work at high temperatures and pressures generating hazardous compounds derived from sugar degradation [3]. For this reason the search of novel pretreatments able to disrupt cell walls which render more available cellulose and hemicellulose without inhibitors for the subsequent enzymatic hydrolysis is relevant [4]. Thermoplastic extrusion is a promising technique for the biomass processing to bioethanol production [1, 5]. Extrusion provides a continuous reaction system in which the feedstock is effectively mixed, compressed, melted, and plasticized at the barrel end changing the raw material's physical-chemical properties. This high productivity process employs short residence time and is easily adaptable and scalable [4]. In addition, extrusion does not generate solids losses nor hazardous byproducts and effluents and has an efficient water use [6]. The twin-screw extruders have shown superiority over single screw counterparts [7] because of their versatility due to the easy build-up of different elements along the screw shaft providing different functionalities [8]. The extrusion parameters like barrel temperature, screws speed, and moisture have remarkable effects in the processing of the raw materials like increasing in the surface area and porosity [3]. The use of enzymatic hydrolysis provides a specific sugar production treatment with mild process conditions and ample sugar yields [2]. The combination of the extrusion and enzymatic treatments provides a process free of unwanted wastes.

The sweet sorghum is an excellent crop for bioethanol production due to its dual capacity of providing a sugar juice (rich in glucose, fructose, and sucrose) and spent lignocellulosic biomass (rich in C5 and C6 sugars) that are efficiently converted into first and second-generation ethanol [9]. The integral use of these feedstocks enables the possibility of a reduction of the ethanol production cost [10]. The objective of this research was to determine optimum extrusion parameters (last zone of the barrel temperature, screws speed, and feedstock moisture content) of sweet sorghum bagasse for its subsequent sugar enzymatic hydrolysis and fermentation with the strain* Issatchenkia orientalis* 20381 into bioethanol.

## 2. Materials and Methods

### 2.1. Materials

Sweet sorghum (*Sorghum bicolor* (L.) Moench) bagasse was procured from the research plots of the Instituto Nacional de Investigaciones Forestales, Agrícolas y Pecuarias (INIFAP) C.E., located in Celaya, Guanajuato, at 1760 m above sea level (coordinates 20°34′47′′N, 100°49′13′′W). After mechanical juice extraction, the bagasse was transported to the Tecnológico de Monterrey, Monterrey Campus, and dried at 50–60°C for 24 h. The dry bagasse was ground in a knife mill (Wiley Mill, Swedesboro, NJ) equipped with a 1 mm sieve.

### 2.2. Chemical Characterization

Moisture was determined using the AACC standard assay 44–15. For the structural carbohydrates assay the sweet sorghum bagasse (SSB) was washed of any soluble component in water or ethanol according to the methods recommended by the National Renewable Energy Laboratory (NREL) [11]. Then, the insoluble fiber was hydrolyzed and filtered for HPLC analysis as recommended by Sluiter et al. [12].

### 2.3. Extrusion Pretreatment

A twin-screw corotating extruder (BTSM-30, Bühler AG, Uzwil, Switzerland) with a barrel composed of 5 zones and two independent feeders for the solid raw material and water was used. The temperature of the fifth zone of the barrel was controlled by a heat exchanger device (Tool Temp, Bühler AG, Uzwil, Switzerland). The total length and outer diameter of the screws were of 800 mm and 30 mm, respectively, and the L/D ratio was 20. A die with a single 4 mm hole was used. The screws configuration was composed of three different sections: inlet/conveying elements section (for the introduction and transport of the dry feedstock and water), mixing elements section, and the final work elements section composed for kneading and reverse elements.

#### 2.3.1. Experimental Design and Extrusion Conditions

A central composite design was used. Three different factors were evaluated: conditioning moisture content, screws speed, and temperature applied in the last section of the extruder barrel. Each independent variable had two levels: 30 or 50% moisture, 100 or 200 rpm, and 50 or 100°C, respectively. A center point with the conditions 40% moisture, 150 rpm, and 75°C was employed. Four center points were performed and three replicates were used for each design point. The solid feed rate was set constant at 5.7 kg·h^−1^ for all conditions.

### 2.4. Enzymatic Saccharification

The biocatalysis assays were made with a total volume of 100 mL in 500 mL flasks using 10% solid bagasse fraction for the central composite design and 10, 12, 14, 16, 18, 20, 22, 24, 26, or 28% solids for the loading assays. Citrate (50 mM) buffer adjusted to pH 5 with 10 mM of sodium azide was used for all hydrolyses. Biocatalyses were carried out in an orbital shaking incubator (VWR Model 1575) set at 50°C and 150 rpm for 24, 48, 72, or 96 h of reaction time. For all other assays, 72 h reaction time was employed. Novozymes enzymes NS22086, NS22083, NS22118, NS22119, NS22002, and NS22035 and dosages of 5, 0.25, 0.6, 0.4, 2, and 0.06% with respect to the solids loading were used, respectively. These enzymes consist in a fibrolytic cocktail of *β*-glucosidase, *β*-glucanase, arabinase, hemicellulase, pectinase, xylanase, and glucoamylase. The declared activity for each enzyme was 1,000 endoglucanase units (EGU)/g, 250 cellobiase units (CBU)/g, 100 fungal *β*-glucanase units (FBG)/g (13,700 polygalacturonase units (PGU)/g), 2,500 fungal xylanase units (FXU-S)/g, 45 FBG/g, and 750 PGU. The dosages used were 5.00, 0.60, 0.40, 0.25, 2.00, and 0.06% w/w total solids, respectively.

### 2.5. Fermentation Process

The fermentation was performed with the strain* Issatchenkia orientalis* ATCC 20381 in 500 mL Erlenmeyer flasks with 200 mL of the hydrolysates by triplicate. Beforehand, the strain was incubated in Difco YM broth (Becton, Dickinson and Company, USA) in an orbital shaking incubator (VWR Model 1575) set at 28°C and 100 rpm. The hydrolysates were adjusted to 12% solids with citrate buffer pH 5. A concentration of 1 × 10^6^ cells·mL^−1^°Brix^−1^ was inoculated into each prepared reaction flask. Aliquots of the fermentation broth were taken at 0, 12, 24, 48, and 72 h of fermentation. The aliquots were centrifuged at 4500 rpm and filtered through a 0.22 *μ*m filter. The total sugars, inhibitors, and ethanol of the filtered samples were analyzed by HPLC while the free amino nitrogen (FAN) was determined after reaction with ninhydrin. The amount of FAN was determined in a spectrophotometer set at 420 nm. The calibration curve was constructed using glycine as standard [13].

The fermentation was performed without any nutrient supplementation at 28°C in an incubator (VWR Model 1575) under anaerobic conditions.

### 2.6. HPLC Quantification of Sugars and Inhibitors

The enzymatic saccharified samples were treated as described in previous research [14]. Analytes were separated by a Shodex SH1011 column (300 × 7.8 mm) with a flow rate of 0.6 mL per minute of HPLC-grade water containing 5 mM H_2_SO_4_ for the quantification of inhibitors and ethanol. The sugar quantification was performed with a Shodex SP0810 column and a cation/anion deasher (Biorad). The column temperature was set at 60 and 85°C for inhibitors and sugars, respectively. Also, the detector was at 50°C and the autosampler (refractive index detector Waters 2414) was at 4°C. Standards of ethanol, cellobiose, D-glucose, D-xylose, L-arabinose, D-mannose, D-galactose, acetic acid, 5-hydroxymethylfurfural, and furfural (Sigma Chemical Co., St. Louis, MO) were used. The run times for sugars and inhibitors quantifications were 20 and 45 min, respectively.

### 2.7. Calculations

The sugar yields were expressed per gram of hydrolyzed sweet sorghum bagasse. The calculations for total and individual sugar yields and recoveries were the following:

(1) Yi=Si×VTXT,YT=∑Yi,Ri=YiBi×100,Bi=Glucans,mannans,or  galactans×1.11,and  arabinans  or  xylans×1.14,RT=YTBT×100,EY=E×VTXT,RE=EYYT×100,EV=EYρE,

where *Y*_*i*_ = individual sugar yield [mg·g^−1^], *S*_*i*_ = individual sugar detected with HPLC [mg·mL^−1^], *V*_*T*_ = total volume recovered [mL], *X*_*T*_ = total hydrolyzed solids [g], *Y*_*T*_ = total sugars [mg·g^−1^], *R*_*i*_ = individual sugar yield recovered [%], *B*_*i*_ = individual structural sugar per gram of sweet sorghum bagasse [mg·g^−1^], *R*_*T*_ = total sugars yield recovered [%], *B*_*T*_ = total structural sugars per gram of sweet sorghum bagasse [mg·g^−1^], *E*_*Y*_ = ethanol yield [mg·g^−1^] per gram of bagasse, *E*_*V*_ = ethanol volume [mL·g^−1^], *ρ*_*E*_ = ethanol density [0.789 g·mL^−1^], *E* = ethanol detected with HPLC [mg·mL^−1^], and *R*_*E*_ = ethanol yield [%].

### 2.8. Statistical Analysis

The analysis of data generated by the central composite design and ANOVA were performed with software Minitab 14 with a statistical significance (*α*) of 0.05 to determine significant differences between treatments and the correlations coefficients (*R* ^2^). For the enzymatic and fermentation data a *t*-test were used and means were compared using the Fisher's exact tests. The response surfaces were plotted with the Statgraphics Centurion XVI software.

## 3. Results and Discussion

### 3.1. Chemical Characterization

The sweet sorghum bagasse chemical composition was the following: 31.48% of glucans, 9.33% of xylans, 0.94% of mannans, 2.94% of arabinans, 3.37% of galactans, 25.42% of water extractives, 7.79% of ethanol extractives, 0.07% of acetyl groups, 4.90% of acid ashes, and 13.03% of total lignin. This meant that the SSB contained about 50% fiber components (537.4 mg of total sugars per gram of dry bagasse). The high lignin content is a strong barrier for the cellulose and hemicellulose accessibility [2]. Shen et al. [10] reported SSB with 10% more sugars, but 5% more lignin than the counterpart tested herein. Cao et al. [15] reported SSB with 49.78% cellulose and 27.72% hemicellulose. This feedstock also contained higher amounts compared with that SSB used in this research. However, although our SSB had low glucose concentration, its low lignin content could be advantageous for the extrusion pretreatment because it has been shown that lignin decreases the pretreatment efficiency and acts as a physical barrier against chemicals and enzymes [16, 17]. These differences were probably due to variety differences, agronomical conditions, and crop maturation.

### 3.2. Extrusion Effect

#### 3.2.1. Release of Total Sugars

**Figure 1:**
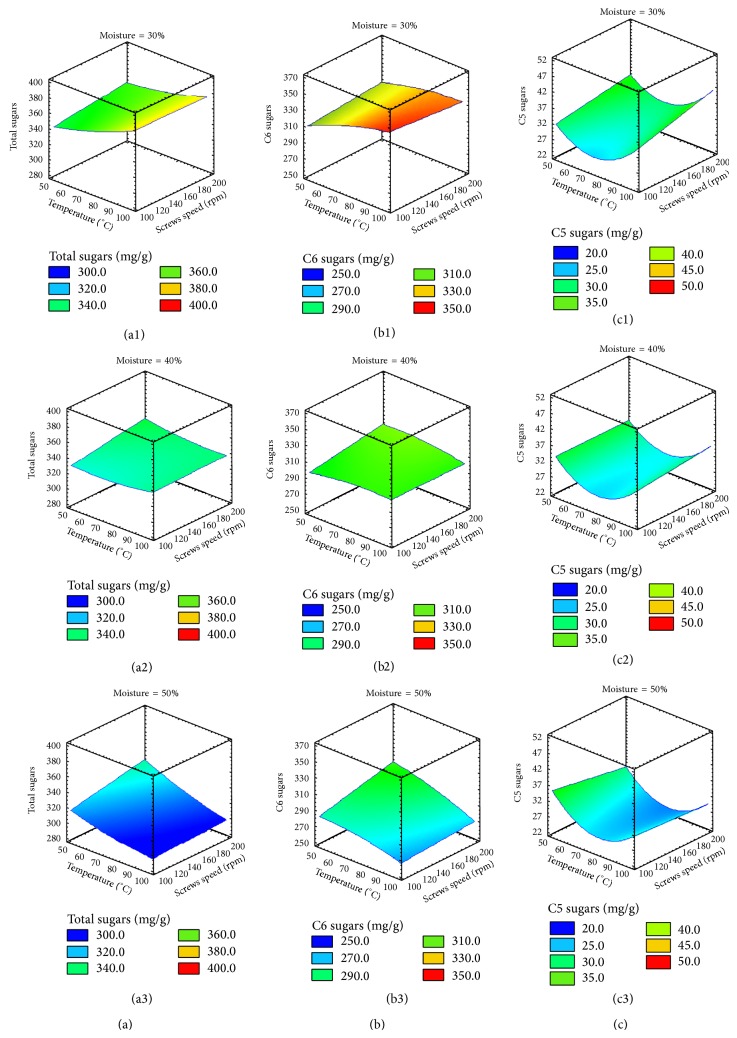
Response surfaces for total sugars ((a1), (a2), and (a3)), C6 ((b1), (b2), and (b3)), and C5 ((c1), (c2), and (c3)) generated from sweet sorghum bagasse extruded at different temperatures (50, 75, or 100°C), screws speeds (100, 150, or 200 rpm), and moistures inside the barrel (30, 40, or 50%) hydrolyzed with fiber degrading enzymes expressed in sugars milligrams per gram of bagasse (dw).

A high linear correlation coefficient of 93.1 was obtained for the total sugars with respect to temperature, screws speed, and moisture (Figures 1(a1), 1(a2), and 1(a3)). The moisture content and the interaction between temperature and moisture were the most significant extrusion parameters with a *P* value = 0.000 followed by the screws speed with a *P* value = 0.013. As expected, the amounts of total free sugars as well as the C5 and C6 sugars concentrations were strongly affected by the extrusion parameters. Higher sugar yields were obtained at low tempering moisture, high screws speeds, and high temperature (Figure 1(a1)). The crystalline cellulose structure represents a strong barrier for the active sites of enzymes [5]. In this nonchemical pretreatment, the extruder shear stress was the mean way of action to enhance the decrystallization of cellulose and its porosity and susceptibility to fibrous degrading enzymes. The tempering moisture of the feedstock was the main factor affecting disruption of fiber components because it directly affected the shear stress rate [3]. When the tempering moisture was increased, the SSB ran more easily through the extruder. Thus, with higher moistures the shear stress was reduced decreasing the susceptibility of the extruded feedstock for the subsequent enzymatic hydrolysis step. The extruder temperature and the screws speeds also affected the yield of individual sugars specially those belonging to the C5 group (Sections 3.2.2 and 3.2.3). It was observed that at high extruder temperatures a lower feedstock viscosity inside the barrel was obtained [3], and this effect improved when the speed of the screws was increased. Karunanithy et al. [18] extruded different varieties of switchgrass, big bluestem, and prairie corn finding a better glucose recovery after enzyme treatment when extruding the feedstocks at 200 rpm in a single screw extruder. In this research, the optimum extruding conditions were 30°C, 200 rpm screws speed, and 30% feedstock moisture content. With these conditions it was possible to release the maximum amounts of sugar after the subsequent enzyme treatment. Approximately 70.4% of the sugars were released from the fiber matrix. Thus, the extruded feedstock processed under the conditions mentioned above was further converted into fermentable sugars and ethanol. The utilization of higher screws speeds (400 or 500 rpm) decreased sugar recovery to only 52.2 and 58.6%, respectively. According to Karunanithy and Muthukumarappan [6], the heat produced from the higher screws speeds affected negatively the viscosity of the feedstock inside the barrel and in some instances caused the blocking of the extruder. The same author [3] obtained an overall yield of 44.5% sugars associated to cell walls of switchgrass without the generation of inhibitory compounds when operating a single screw extruder at 50 rpm, 150°C, and 15% moisture content. Karunanithy et al. [18] employed 4 heaters to maintain the barrel temperature and increase the efficiency of transformation of lignocellulose materials. In this investigation, only one heat exchanger was used to maintain the barrel at high temperature. Only the last section of the extruder was heated because the screw configuration was designed to cause friction and intrinsic heat to properly treat the SSB. Regarding the extruder specific mechanical energy (SME) Lamsal et al. [5] obtained good sugar recoveries in a range of 222 up to 639 Wh·kg^−1^. The SME at 100, 150, and 200 rpm were 266.8, 276.0, and 314.8 Wh·kg^−1^ when extruding 30% tempered SSB, respectively. The results indicated that the thermoplastic extrusion was an adequate continuous pretreatment for second-generation bioethanol production without the generation of hazardous compounds that decrease the efficiency of fermentation of hydrolyzed lignocellulosic feedstocks.

#### 3.2.2. Release of C6 Sugars

After the enzymatic hydrolysis, it was possible to release significant amounts of cellobiose, glucose, galactose, and mannose from the extruded SSB. The mathematical models for C6 sugars were linear with correlation coefficients greater than 85% (Figures 1(b1), 1(b2), and 1(b3)). Almost all the factors and their interactions had significant effects over the efficiency of the subsequent step of enzymatic hydrolysis. For cellulose hydrolysis, all the independent factors, double and triple interactions, had significant effects (*P* value ≤ 0.009). The same effects were observed with galactose with the exception that the feedstock moisture content did not have a significant effect. On the other hand, for glucose and mannose all the effects and interactions were statistically significantly different except for temperature (*P* value ≤ 0.010). Karunanithy and Muthukumarappan [3] used a single screw extruder to pretreat switchgrass and found that at 150 rpm there was a better sugar release. Also, these researchers found that at 100°C and low tempering moisture contents the glucose recovery was higher. Although not all the extrusion parameters were significant for every sugar released, all these variables needed to be optimized in order to have an efficient pretreatment. The interaction between extrusion parameters allowed the efficient C6 sugars release from SSB.

#### 3.2.3. Release of C5 Sugars

A linear correlation was obtained for the enzymatic release of arabinose with *R* ^2^ = 72.63%. The main effects were the moisture content and the interactions temperature-screw speed and screws speed-feedstock moisture content (*P* values ≤ 0.002). On the other hand, the enzymatic release of xylose fitted as a quadratic correlation (*P* value = 0.000) with *R* ^2^ = 74.27%. The main extrusion effects for xylose hydrolysis were the square temperature and the interactions temperature-moisture, screws speed-moisture, and temperature-screws speed-moisture content. The square temperature and the screws speed-moisture interactions had a *P* value = 0.037, which meant less significance than the other values that had a *P* value = 0.000. The modeling of both C5 sugars fitted as a quadratic correlation temperature dependent (*R* ^2^ = 51.72, *P* value = 0.010). The arabinose linear modeling was overshadowed by the quadratic effect of the xylose modeling mainly for the higher amount of this sugar compared to arabinose. Karunanithy et al. [18] found that the temperature had a positive effect on the xylose release from diverse lignocellulosic raw materials. Similar to the C6 sugars, it was found that the feedstock moisture and the extrusion parameters interactions had a positive effect over the SSB enzymatic hydrolysis. Furthermore, the feedstock moisture had a direct effect over the temperature due to the shear stress effect. The heat is a very important condition for hemicellulose conversion to sugars [2]. The use of higher feedstock moisture contents reduced the heat exchange and therefore sugars yields [3].

Even if the arabinose had a correlation coefficient similar to that of xylose, the global behavior of C5 enzymatic release had a quadratic correlation (Figures 1(c1), 1(c2), and 1(c3), resp.). As discussed independently for xylose and arabinose, the feedstock moisture content was the main factor affecting the C5 sugar release. A low feedstock moisture yielded more sugars. On the other hand, at higher moistures (40 and 50%) the sugars concentrations were reduced significantly, especially when the extruder was operated at screws speeds higher than 100 rpm. Also, the interaction of temperature with other effects was important specifically for the susceptibility of hemicellulose to enzymatic hydrolysis. The quadratic effects had a strong influence in the center conditions when the C5 sugars concentrations drop to lower concentrations. The effect of the significant interactions was positive for sugars recovery, especially at 30% moisture content, 100°C, and 200 rpm. The opposite effect was obtained at 50% feedstock moisture content, where the sugar concentrations with 100°C and 200 rpm were significantly less compared to 50°C and 100 rpm (Figure 1(c1)). Despite this specific effect, the C5 sugar concentrations remained higher using a feedstock moisture content of 30%.

#### 3.2.4. Inhibitors Compounds

As expected, any enzymatic hydrolysate did not contain significant amounts of acetic acid, furfural, or HMF. These inhibitors are produced in large quantities in fiber feedstocks pretreated with acid or alkaline chemicals. Karunanithy et al. [18] and Karunanithy and Muthukumarappan [3] reported slight amounts of acetic acid in some of their enzymatically saccharified lignocellulosic extruded materials. The acetic acid is generated from the disruption of the hemicellulose [1, 19]. The hydrolysates obtained in this research did not contain acetic acid, indicating that the heat applied during extrusion was not enough to release the acetic groups from lignocellulose. Furfural and HMF are produced from the C5 and C6 sugars dehydration [20] after chemical pretreatments. The residence time of the feedstock and the temperatures inside the extruder were not enough to degrade sugars into these types of inhibitors [7].

### 3.3. Enzymatic Studies

#### 3.3.1. Enzymatic Saccharification Time Effect

**Figure 2:**
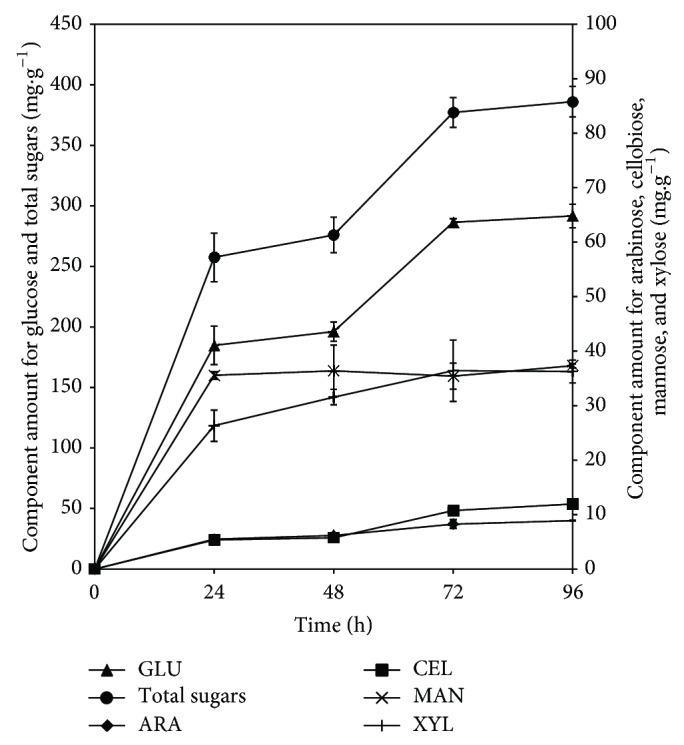
Sugars generated from sweet sorghum bagasse extruded at 30% moisture, 200 rpm, and 100°C at 10% solids loading after 24, 48, 72, and 96 h treatment with fiber degrading enzymes.

The major glucose and total fermentable sugar release occurred after 24 h hydrolysis. At this point in time 52.8% of the glucose and 47.9% of the total sugars were released from the extruded SSB (Figure 2). There were no significant differences between the total amounts of sugars at 24 and 48 h of enzymatic saccharification. In the case of the xylose concentration, there were no significant differences after 48 h of hydrolysis. The mannose reached its maximum concentration after 24 h hydrolysis with no significant differences throughout the proposed biocatalysis step. The amounts of sugars were not significantly different when hydrolysates produced after 72 and 96 h of saccharification were compared. Regarding glucose, the maximum concentration was reached at 72 h where 82% of the total available glucose was produced. Arabinose was the only sugar which reached the maximum concentration after 96 h of hydrolysis. Even if the major amount of sugars was released during the first 24 h, hydrolyses times between 48 and 72 h were necessary to release the remaining 22% of the total sugars. During the saccharification, the hydrolyzed sugars block the active site of the enzymes [21]. For this reason, the SSB hydrolysis had an apparent time of slow enzymatic activity between the 24 and 48 h reactions. According to Pandey [1] cellulases are inhibited approximately 75% when the glucose concentration reached 3 g·L^−1^. After 24 h of enzymatic hydrolysis the hydrolysates contained 18.47 g·L^−1^ of glucose, enough amount to produce cellulase inhibition. There were no significant differences in total sugars concentrations when hydrolysates generated after 72 and 96 h were compared. Thus, 72 h of biocatalysis was selected as the best hydrolysis time. At this time 73% of the total sugars were released.

Shen et al. [10] reported maximum sugars concentrations after 96 h enzymatic hydrolysis using SSB that was previously steam-pretreated. These particular conditions only achieved 68% of glucose hydrolysis. A previous research that tested the use of thermoplastic extrusion of SSB tempered to 30% moisture [22] obtained 67.5% of total sugars recovery. The feedstock was extruded at 150°C and 100 rpm and then saccharified using 5% bagasse loading. With the new extrusion conditions it was possible to hydrolyze 70% sugars using the double amount of SSB.

#### 3.3.2. Solids Loading Assay

**Figure 3:**
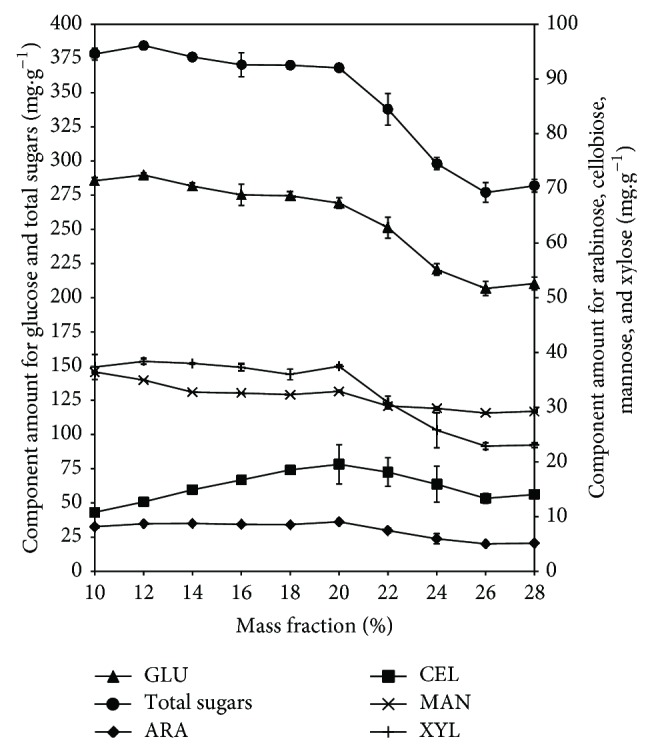
Sugars generated per g bagasse (dmb) at 10, 12, 14, 16, 18, 20, 22, 24, 26, or 28% loading of sweet sorghum bagasse extruded at 30% moisture, 200 rpm, and 100°C and subsequently treated with fiber degrading enzymes for 72 h.

The effects of different mass loading fractions in the enzymatic hydrolysis performance of the extruded sweet sorghum bagasse are depicted in Figure 3. Xylose and arabinose were detected in significant amounts whereas galactose was not generated during the biocatalysis step. There were no statistical differences between total sugars concentrations when the 10 and 20% mass fractions were compared. It was possible to hydrolyze around 70% of the total sugars at the loading of these solids. Xylose and arabinose were generated similarly during the proposed enzyme hydrolysis. The maximum concentrations were observed in the 10% mass hydrolysates and remained with no significant change when compared with hydrolysates elaborated with 12, 14, 16, 18, or 20% mass fractions. At 22% or higher solids, the C5 sugars concentration decreased although the detrimental effect was not observed in the 26 and 28% bagasse fractions. Mannose reached its higher concentration in hydrolysates containing 10 to 20% mass loading. After these solid loadings there were no significant differences when 14, 16, 18, or 20% mass loadings were compared. The mannose in hydrolysates produced with 22% solids maintained the same average concentration compared to the rest of the higher solid mass fractions tested. Regarding cellobiose, it increased constantly in hydrolysates produced with 10 to 20% mass loadings. The maximum concentrations (18 mg·g^−1^ bagasse) were achieved in hydrolysates containing 18 to 24% bagasse loadings. At higher solid concentrations cellobiose decreased to values under 15 mg·g^−1^ bagasse. The increase in cellobiose concentration was related to the reduction in glucose concentrations. The accumulation of cellobiose likely occurred due to its product inhibitory effect on endoglucanases, exoglucanases, and *β*-glucosidases [18, 23]. When more solids were used, higher amounts of cellobiose were released and enzymes inhibited yielding lower amounts of sugars especially in hydrolysates containing 22% solids. The maximum glucose concentration was achieved at 10% SSB loading. After this concentration there were no significant differences between the group of hydrolysates containing 12, 14, 16, or 18% solids and the other group containing 16, 18, or 20% SSB solids. The glucose concentrations in hydrolysates containing 22, 24, or 26–28% were significantly different. The hydrolysate containing 20% extruded bagasse loading was not significantly different compared to the counterpart containing 10% solids. The proposed thermoplastic extrusion pretreatment favored cellulose decrystallization and increased the surface area and porosity of the feedstock increasing the amount of soluble fiber [6, 8]. Due to these effects it was possible to increase the bagasse loading during the fiber degrading enzymatic step. The hydrolysate containing 10% SSB solids contained 37.8 g·L^−1^ of total sugars whereas its counterpart at 20% solids achieved 73.6 g·L^−1^. Therefore, the hydrolysate with 20% solids contained almost twice the sugar amount compared to the 10% solid counterpart. It was possible to employ high solid concentrations with extruded SSB without seriously affecting enzymes performance. However, the use of higher mass fractions did not further improve sugar generation. Although the extrusion pretreatment improved the subsequent enzymatic hydrolysis, the water to solid ratio became a limiting factor in hydrolysates containing more than 20% solids. The role of free water is relevant because it acts as solvent enhancing the contact between substrate and enzymes. Also the water has a direct effect over the viscosity and the enzyme mass transfer in the hydrolysates [21]. The use of higher than 20% extruded SSB loadings decreased free water necessary to enhance enzyme performance and sugars yields. In addition, the cellobiose amounts increased indicating the likely enzymatic inhibition by products. These results indicated that the 20% mass fraction achieved high sugars yield using relatively lower amounts of water. This particular hydrolysate contained 7.3% total sugars suited for the following fermentation step. Despite the chemical composition, the enzymatically saccharified extruded SSB using a 20% mass loading contained 10% more total sugars compared to counterparts produced by Shen et al. [10].

### 3.4. Extruded Sweet Sorghum Bagasse Fermentation

There were no significant differences between the sugar consumption and ethanol generation in hydrolysates with and without insoluble solids (Table 1). Although all the soluble sugars were metabolized by the yeast, the main carbohydrate source was glucose. After the first 12 h fermentation, 23.6 and 32.2% of the total sugars were consumed in hydrolysates free of insoluble solids and counterparts containing insoluble solids. After 24 h fermentation, 99.4 and 96.5% of the glucose were consumed in hydrolysates with and without insoluble solids, respectively (Table 1). During the first 12 h fermentation, only 23.6% of the total sugars in hydrolysates containing insoluble solids content were consumed. This particular system generated 21.78 mg ethanol·g^−1^ extruded bagasse. For the fermentation of the counterpart free of insoluble solids, 32.2% of the total sugars were consumed at the first 12 h. The arabinose was not detected after 12 h in both fermenting broths, which indicated the effective consumption of this sugar by the* I. orientalis* strain. On the other hand, 25% of the available xylose was consumed after 12 h fermentation in hydrolysates containing insoluble solids and this sugar was not further fermented during the posterior 60 h fermentation. Similarly, the fermentation of hydrolysates without insoluble solids consumed 25.6% of the total xylose present in the broth. The low and high xylose and glucose consumptions were previously documented [22]. Galactose was metabolized slowly along the fermentation of hydrolysate with insoluble solids. On the other hand, the fermented counterpart without solids consumed all the galactose after 48 h biocatalysis. After two days of fermentation, the sugars consumed were transformed into 200 Ml ethanol·g^−1^ SSB in hydrolysates with and without insoluble solids, respectively. These ethanol conversions efficiencies were 40.9 and 41.4%, respectively. There were no significant differences in ethanol concentrations when 24 and 48 h fermentation times were compared. After 48 h fermentation, the yeast only used the remaining sugar to survive and did not generate significant amounts of ethanol. In both cases, the mannose was not used and therefore it increased its concentration in both fermented broths. Interestingly, in both types of fermentation, the cellobiose decreased with the reaction time, but the fermentation in presence of insoluble solids decreased significantly after 24 h. Probably the* I. orientalis* had a cellulosic enzymatic activity with solids presence. Regarding the alpha amino nitrogen consumption, it followed a similar trend compared to sugars consumption. During the first 12 h fermentation, only 5.6 and 4.1% of FAN were consumed in hydrolysates with and without insoluble solids. However, FAN consumption increased to 50% after 24 h fermentation. This behavior together with the glucose consumption marked the final stage of the fermentation. Only hydrolysates without insoluble solids kept producing significant amounts of ethanol and consumed FAN during the last stage of the programed fermentation. This indicates that the* I. orientalis* metabolized these components for survival in the absence of glucose.

Fermentation of enzymatically saccharified extruded SSB adjusted to 12% solids generated 19.20 and 18.53 g of ethanol·L^−1^ in hydrolysates prepared with and without insoluble solids, respectively. Shen et al. [10] obtained similar yields of 19.8 g etnaol·L^−1^ after fermenting enzymatically saccharified steam pretreated SSB with different strains of* S. cerevisiae*. Choi et al. [4] performed simultaneous saccharification and fermentation of rapeseed straw pretreated with H_2_SO_4_ in a twin-screw extruder obtaining 16.01 g of ethanol per L. The combination of diluted acid pretreatment and extrusion achieved a good ethanol yield, but the process recommended the washing of the chemically and mechanically treated feedstock to remove inhibitors.

The efficacy of the physical pretreatment proposed herein without the use of chemicals is ideally suited to the subsequent saccharification step and yields hydrolysates with high amounts of potentially fermentable C5 and C6 sugars without inhibitors that are effectively fermented into bioethanol by yeast strains such as* I. orientalis*. In this research, hydrolysates were diluted due to the utilization of a regular fermentation strain. The utilization of an osmotolerant yeast will likely make it possible to ferment without diluting. Assuming that the sweet sorghum cultivar yields around 85 ton per hectare [9] with 50% of sweet juice it is possible to convert the spent lignocellulose biomass into 4309 L·Ha^−1^ of ethanol. Thus, the integral use of sweet sorghum to generate first-generation ethanol from the sweet juice and second-generation ethanol from bagasse processed by the technology proposed herein can yield more than 7369 L bioethanol·Ha^−1^.

## 4. Conclusions

Extrusion was a viable pretreatment for second-generation bioethanol using SSB because it did not generate inhibitors and enhanced the subsequent enzymatic conversion into fermentable sugars. The SSB extruded at 100°C and 200 rpm and with 30% moisture generated up to 70% of the total sugars after treatment with fiber degrading enzymes. After 72 h enzyme hydrolysis and using 20% mass fraction 73.6 g·L^−1^ total sugars were obtained. Fermentation of these hydrolysates yielded about 200 mL ethanol·kg^−1^ of extruded SSB. Interestingly, it was not necessary to remove the insoluble solids of the enzymatically treated SSB hydrolysate for a successful fermentation.

## Figures and Tables

**Table 1:** Different sugars and ethanol concentrations from extruded sweet sorghum bagasse at 100°C, 200 rpm, and 30% moisture, fermented with *Issatchenkia orientalis* 20381 at 12% solids fraction after 72 h enzymatic saccharification^1,2^.

Compound [mg·g^−1^]	*Issatchenkia orientalis* ATCC 20381 at 12% solids loading ferementation					*Issatchenkia orientalis* ATCC 20381 without solids				
	0 h	12 h	24 h	48 h	72 h	0 h	12 h	24 h	48 h	72 h
Arabinose	4.20 ± 0.08	ND	ND	ND	ND	4.26 ± 0.07	ND	ND	ND	ND
Cellobiose	3.94 ± 0.27	12.29 ± 2.32	12.07 ± 0.06	6.30 ± 0.25	2.75 ± 0.05	3.31 ± 0.15	12.84 ± 0.36	12.92 ± 0.36	10.41 ± 0.13	9.88 ± 0.77
Galactose	3.71 ± 0.28	2.90 ± 0.12	1.51 ± 0.15	0.47 ± 0.01	0.16 ± 0.01	3.69 ± 0.06	0.27 ± 0.04	ND	ND	ND
Glucose	337.01 ± 1.10	250.78 ± 13.76	2.00 ± 0.35	1.48 ± 0.44	ND	326.92 ± 4.25	213.83 ± 5.26	11.45 ± 3.96	ND	ND
Mannose	ND	1.22 ± 0.08	2.30 ± 0.19	2.70 ± 0.03	3.09 ± 0.05	2.07 ± 0.03	1.46 ± 0.08	1.63 ± 0.17	2.01 ± 0.01	2.03 ± 0.19
Xylose	36.80 ± 0.29	27.60 ± 0.53	28.30 ± 0.53	26.61 ± 0.22	23.39 ± 2.12	32.51 ± 0.18	24.16 ± 1.03	28.45 ± 1.82	26.96 ± 1.70	22.23 ± 0.91
Total sugars	385.66 ± 6.79	294.79 ± 11.71	45.90 ± 0.48	37.56 ± 0.01	29.39 ± 2.05	372.76 ± 4.74	252.60 ± 6.77	54.44 ± 1.12	39.37 ± 1.56	31.14 ± 1.87
Ethanol	ND	21.78 ± 1.10	160.06 ± 3.10	157.89 ± 3.03	147.63 ± 1.81	ND	16.94 ± 0.49	154.34 ± 3.24	156.36 ± 1.15	127.63 ± 5.66
Free amino nitrogen^3^	98.65 ± 0.95	93.13 ± 4.00	49.46 ± 4.76	47.57 ± 3.24	47.30 ± 0.95	144.88 ± 0.38	138.81 ± 3.43	75.74 ± 1.52	75.45 ± 1.72	50.94 ± 3.81

^1^The compounds are in milligrams of sugar per gram of sweet sorghum bagasse dry basis.

^2^The compounds not detected by the HPLC were reported with the acronym ND.

^3^The free amino nitrogen is in milligrams per liter.

## References

[1] Pandey A. (2011). *Biofuels: Alternative Feedstocks and Conversion Processes*.

[2] Bensah E. C., Mensah M. (2013). Chemical pretreatment methods for the production of cellulosic ethanol: technologies and innovations. *International Journal of Chemical Engineering*.

[3] Karunanithy C., Muthukumarappan K. (2010). Effect of extruder parameters and moisture content of switchgrass, prairie cord grass on sugar recovery from enzymatic hydrolysis. *Applied Biochemistry and Biotechnology*.

[4] Choi C. H., Kim J. S., Oh K. K. (2013). Evaluation the efficacy of extrusion pretreatment via enzymatic digestibility and simultaneous saccharification & fermentation with rapeseed straw. *Biomass and Bioenergy*.

[5] Lamsal B., Yoo J., Brijwani K., Alavi S. (2010). Extrusion as a thermo-mechanical pre-treatment for lignocellulosic ethanol. *Biomass and Bioenergy*.

[6] Karunanithy C., Muthukumarappan K. (2011). Influence of extruder and feedstock variables on torque requirement during pretreatment of different types of biomass—a response surface analysis. *Biosystems Engineering*.

[7] Lin Z., Liu L., Li R., Shi J. (2012). Screw extrusión pretreatments to enhance the hydrolysis of lignocellulosic biomass. *Journal of Microbial & Biochemical Technology*.

[8] Moscicki L. (2011). *Extrusion-Cooking Techniques: Applications, Theory and Sustainability*.

[9] Serna-Saldivar S. O., Chuck-Hernandez C., Pérez-Cariillo E., Heredia-Olea E., Pinheiro Lima M. A., Policastro Natalense A. P. (2012). Chapter 3: sorghum as a multifunctional crop for the production of fuel ethanol: current status and future trends. *Bioethanol*.

[10] Shen F., Hu J., Zhong Y., Liu M. L. Y., Saddler J. N., Liu R. (2012). Ethanol production from steam-pretreated sweet sorghum bagasse with high substrate consistency enzymatic hydrolysis. *Biomass & Bioenergy*.

[11] Sluiter A., Ruiz R., Scarlata C., Sluiter J., Templeton D. (2008). *Determination of Extractives in Biomass: Laboratory Analytical Procedure (LAP)*.

[12] Sluiter A., Hames B., Ruiz R. (2008). *Determination of Structural Carbohydrates and Lignin in Biomass. Laboratory Analytical Procedure (LAP)*.

[13] Wright D. W. (1923). The determination of free amino nitrogen in proteins. *The Journal of Biological Chemistry*.

[14] Heredia-Olea E., Pérez-Carrillo E., Serna-Saldívar S. O. (2012). Effects of different acid hydrolyses on the conversion of sweet sorghum bagasse into C5 and C6 sugars and yeast inhibitors using response surface methodology. *Bioresource Technology*.

[15] Cao W., Sun C., Liu R., Yin R., Wu X. (2012). Comparison of the effects of five pretreatment methods on enhancing the enzymatic digestibility and ethanol production from sweet sorghum bagasse. *Bioresource Technology*.

[16] Scheper T., Olsson L. (2007). *Advances in Biochemical Engineering/Biotechnology*.

[17] Sun Y., Cheng J. (2002). Hydrolysis of lignocellulosic materials for ethanol production: a review. *Bioresource Technology*.

[18] Karunanithy C., Muthukumarappan K., Gibbons W. R. (2013). Effects of extrudr screw speed, temperature, and enzyme levels on sugar recovery from different biomasses. *ISRN Biotechnology*.

[19] Rodríguez-Chong A., Ramírez J. A., Garrote G., Vázquez M. (2004). Hydrolysis of sugar cane bagasse using nitric acid: a kinetic assessment. *Journal of Food Engineering*.

[20] Gámez S., González-Cabriales J. J., Ramírez J. A., Garrote G., Vázquez M. (2006). Study of the hydrolysis of sugar cane bagasse using phosphoric acid. *Journal of Food Engineering*.

[21] Kristensen J. B., Felby C., Jørgensen H. (2009). Yield-determining factors in high-solids enzymatic hydrolysis of lignocellulose. *Biotechnology for Biofuels*.

[22] Heredia-Olea E., Pérez-Carrillo E., Serna-Saldívar S. O. (2013). Production of ethanol from sweet sorghum bagasse pretreated with different chemical and physical processes and saccharified with fiber degrading enzymes. *Bioresource Technology*.

[23] Corazza F. C., Calsavara L. P. V., Moraes F. F., Zanin G. M., Neitzel I. (2005). Determination of inhibition in the enzymatic hydrolysis of cellobiose using hybrid neural modeling. *Brazilian Journal of Chemical Engineering*.

